# Barcoding the* Dendrobium* (Orchidaceae) Species and Analysis of the Intragenomic Variation Based on the Internal Transcribed Spacer 2

**DOI:** 10.1155/2017/2734960

**Published:** 2017-10-17

**Authors:** Xiaoyue Wang, Xiaochen Chen, Pei Yang, Lili Wang, Jianping Han

**Affiliations:** Institute of Medicinal Plant Development, Chinese Academy of Medical Sciences, Peking Union Medical College, Beijing, China

## Abstract

Many species belonging to the genus* Dendrobium* are of great commercial value. However, their difficult growth conditions and high demand have caused many of these species to become endangered. Indeed, counterfeit* Dendrobium *products are common, especially in medicinal markets. This study aims to assess the suitability of the internal transcribed spacer 2 (ITS2) region as a marker for identifying* Dendrobium* and to evaluate its intragenomic variation in* Dendrobium *species. In total, 29,624 ITS2 copies from 18 species were obtained using 454 pyrosequencing to evaluate intragenomic variation. In addition, 513 ITS2 sequences from 26* Dendrobium* species were used to assess its identification suitability. The highest intragenomic genetic distance was observed in* Dendrobium chrysotoxum* (0.081). The average intraspecific genetic distances of each species ranged from 0 to 0.032. Phylogenetic trees based on ITS2 sequences showed that most* Dendrobium* species are monophyletic. The intragenomic and intraspecies divergence analysis showed that greater intragenomic divergence is mostly correlated with larger intraspecific variation. As a major ITS2 variant becomes more common in genome, there are fewer intraspecific variable sites in ITS2 sequences at the species level. The results demonstrated that the intragenomic multiple copies of ITS2 did not affect species identification.

## 1. Introduction

*Dendrobium* is one of the three largest genera of the Orchidaceae family and comprises more than 1,000 species distributed throughout the Asian tropical and subtropical regions as well as Oceania, with 78 species of this genus recorded in China alone [1]. The flowers of* Dendrobium* come in a rich variety of colors and shapes, and in recent years they have increased significantly in commercial value as ornamental flowers. In addition,* Dendrobium* is also well known for its medical value. In fact, one of the earliest records of Orchidaceae plants in ancient Chinese literature is Shen Nong's classic herbal text written approximately 1,500 years ago. Approximately 33 species of* Dendrobium* are used as clinical medications [2], including* Dendrobium officinale*, also known as “Tie Pi Feng Dou,” and* Dendrobium nobile*, also known as “Jin chai shi hu,” as described in the Chinese Pharmacopoeia. Each year, large numbers of* Dendrobium* species are needed for both the flower and medicinal markets. Adulterants and substitutes have become popular in the markets, especially for medicinal purposes. Thus, an effective method of species identification is very necessary.

In eukaryotic genomes, rDNA arrays are often present in hundreds of copies, with copy number varying among different species [3–5]. As a tool to study evolution, the rDNA copy number per genome and sequence variation between species can be used to study phylogenetic relationships and biodiversity [4]. The internal transcribed spacer (ITS) is part of a multicopy gene that encodes ribosomal RNA subunits in all eukaryotic genomes. ITS regions have been used to study biodiversity in bacteria [6], insects [7], marine organisms [8–10], and plants [11], as well as many others. Due to their powerful discriminatory ability and stability among* Dendrobium* species, rDNA sequences have been used for identification and classification purposes [12, 13]. Among the numerous* Dendrobium* species,* D. officinale* has received the greatest amount of attention due to its high medicinal value in China. Ding et al. established a database that included 21* Dendrobium* species labelled “Feng Dou” herbs on the market and proposed that rDNA ITS sequences could be used to identify* Dendrobium* species with high accuracy [14]. Indeed, Zhang et al. accurately identified* D. officinale* from its adulterants using full-length ITS regions [15]. Furthermore, Li et al. performed phylogenetic analyses and identified* Dendrobium* species using rDNA ITS sequences, and their classification based on ITS sequences was identical to traditional classifications for most species [16].

ITS2 is commonly used to infer phylogenetic relationships and has been employed as a DNA barcode for identification purposes. The genes in this region are thought to have evolved in concert, leading to a homogenization of all copies of this gene across the genome [17, 18]. To date, the ITS2 region has been used to identify plants [19–21], fungi [22–24], and insects [25]. Although ITS/ITS2 is extremely useful for both species identification and phylogenetic analyses, it does have drawbacks. One significant problem is the fact that it is present in multiple copies in the genome. Phylogenetic studies typically use consensus sequences that average over all copies in a genome, thereby concealing most intragenomic variation. Indeed, the intragenomic variation and intraspecies divergence in ITS2 present significant challenges for genetic diversity analyses and species identification. In contrast, the evaluation of ITS2 sequences for identification and phylogenetic purposes might prove useful for deep research into intragenomic and intraspecific diversity. While intraspecific divergence in* Dendrobium* has been studied, the issue of intragenomic diversity revealed by multicopy has received increased attention due to the development of next-generation sequencing technology. Here we used pyrosequencing to sequence 18 selected species of* Dendrobium* to perform ITS2 intragenomic diversity analysis. Intra- and interspecific variations among different species were also evaluated using ITS2 sequences in 26 species of* Dendrobium*. Our results indicate that the ITS2 region is a valuable tool for identifying species and analyzing phylogenetic relationships.

## 2. Material and Methods

### 2.1. Sampling, DNA Extraction, PCR Amplification, and Sequencing

Fresh leaves and stems of plants of the genus* Dendrobium* were obtained from different locations (see Appendix S1, in Supplementary Material available online at https://doi.org/10.1155/2017/2734960). Samples were dried at a temperature of 45°C prior to genomic DNA extraction. DNA extraction, PCR amplification, and sequencing were performed as described in previous studies [26, 27]. Approximately 15 mg of dried leaves or 20 mg of dried stems was ground for two minutes (30 revolutions/second) in a FastPrep bead mill (MM400, Retsch, Haan, Germany). DNA was extracted using the Plant Genomic DNA Kit (Tiangen Biotech Co., Beijing, China). Universal primers for the ITS2 region (ITS2F/3R) were used for amplification [27]. Sequencing of the PCR products was performed bidirectionally with the same primers used for the PCR amplification using a 3730XL sequencer (Applied Biosystems, Foster, California, USA). The intragenomic data used were from a previous study by our group [28]. Other sequences were obtained from GenBank (see Appendix S2). Twenty-six* Dendrobium* species with more than ten sequences each were selected for identification analysis.

### 2.2. Data Analysis

ITS2 sequences in this study were subjected to hidden Markov model (HMM) [29] analysis to remove the conserved 5.8S and 26S rRNA genes. Intragenomic and intraspecific Kimura 2-parameter distances were computed using the MEGA 5.2.2 software [30] (default parameters: variance estimation method: bootstrap method; number of bootstrap replications: 1000; model: Kimura 2-parameter model; substitutions to include transition + transversions; rates among sites: uniform rates; and gaps/missing data treatment: complete deletion). Mega 5.2.2 was used to construct a neighbor-joining tree (default parameters: test of phylogeny: bootstrap method; number of bootstrap replications: 1000; model: Kimura 2-parameter model; substitutions to include: transition + transversions; rates among sites: uniform rates; and gaps/missing data treatment: complete deletion).

## 3. Results

### 3.1. Intragenomic Variations in 18 Species of* Dendrobium*

We first investigated the levels of intragenomic variation in the ITS2 regions of 18* Dendrobium* medicinal materials. A summary of the intragenomic variation is provided in Table 1. In total, 29624 ITS2 copies from 18 species were obtained using 454 pyrosequencing in a previous study by our group. The numbers of ITS2 variant copies are 4 to 55, with* D. crepidatum* having the least and* D. chrysotoxum* having the most. Here, we refer to variants with an emergence frequency above 1% as “major variant(s).” Major variants representing more than 90% of ITS2 sequences were found in* D. crepidatum* (p1-1),* D. aphyllum* (P2-1),* D. devonianum* (p3-1),* D. officinale* (p4-1),* D. trigonopus* (p16-1),* D. gratiosissimum* (p15-1),* D. capillipes* (p13-1), and* D. denneanum* (p5-1), which were present at 99.27%, 95.87%, 95.43%, 93.89%, 93.34%, 93.23%, 93.03%, and 91.50% of total sequences, respectively. The major variants of each species were used to calculate the intragenomic genetic distance (IG-GD). The highest IG-GD was found in* D. chrysotoxum* (0.081). In contrast, the genetic distance in five species (*D. officinale*,* D. crepidatum*,* D. aphyllum*,* D. wardianum,* and* D. trigonopus*) was zero, indicating minimal intragenomic diversity.* D. williamsonii* showed the most intragenomic variant patterns of the 18 study species, showing 52 distinct major variant patterns that ranged in prevalence from 25.82% to 1.1%. The GC content of the variants from these 18 species ranged from 47.35% to 56.00%. No obvious differences were observed in the length of major variants.

In this study, the* D. officinale* sample received a total of 2554 reads of 454 pyrosequencing representing ten different variant patterns. The most common major variant represented 93.89% of the ITS2 sequences in the entire genome. After alignment, the consensus sequence of the ten variants from the* D. officinale* genome was 246 bp in length, with 13 variable sites, including two INDELS. The dominant sequence patterns in* D. officinale* were consistent with the sequences obtained via direct PCR sequencing.

### 3.2. Analysis of the ITS2 Region at Intra- and Interspecific Levels

**Figure 1:**
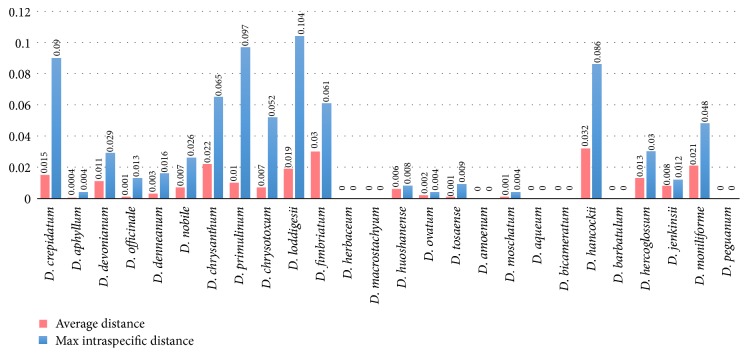
Intraspecific genetic distances from 26* Dendrobium* species revealed by ITS2 region.

In total, 513 ITS2 sequences from 26 species of* Dendrobium* were analyzed for intraspecific genetic distances (IS-GDs). The average IS-GD for each species ranged from 0 to 0.032. The average IS-GD value in seven species (*D. herbaceum*,* D. macrostachyum*,* D. amoenum*,* D. aqueum*,* D. bicameratum*,* D. barbatulum,* and* D. peguanum*) was zero, and the highest average IS-GD value (0.032) was found in* D. hancockii* (Figure 1). The number of variable sites in ITS2 sequences of each species was also calculated (Table 2).* D. officinale* possesses a dominant variant representing 93.89% of the sequences, and out of 61 sequences there were only five intraspecific SNPs. A similar situation was observed in three other species (*D. aphyllum*,* D. devonianum,* and* D. denneanum*), all of which had one dominant ITS2 variant making up more than 90% of sequences. In contrast, there were more variable sites in species where the dominant ITS2 variant made up less than 80% of the sequences. For example, the dominant variant in* D. loddigesii *accounted for only 39.55% of sequences, and this species had 28 intraspecific variable sites. In general, as the dominant ITS2 variant became more common in the genome, there were fewer intraspecific variable sites in ITS2 sequences, with the exception of* D. crepidatum*.

The BLAST1 method, which is based on similarity, was used to assess the reliability of ITS2 sequences for* Dendrobium* species identification. In total, 383 of the 513 ITS2 sequences were correctly identified. The unidentified ITS2 sequences were distributed among six species:* D. officinale*,* D. tosaense*,* D. huoshanense*,* D. moniliforme*,* D. nobile,* and* D. hercoglossum*. The ITS2 sequences of* D. officinale* and* D. tosaense* could not be distinguished using BLAST, and similar issues arose for* D. huoshanense*,* D. moniliforme*,* D. nobile,* and* D. hercoglossum*.

### 3.3. The Neighbor-Joining Tree Based on ITS2 Sequences

**Figure 2:**
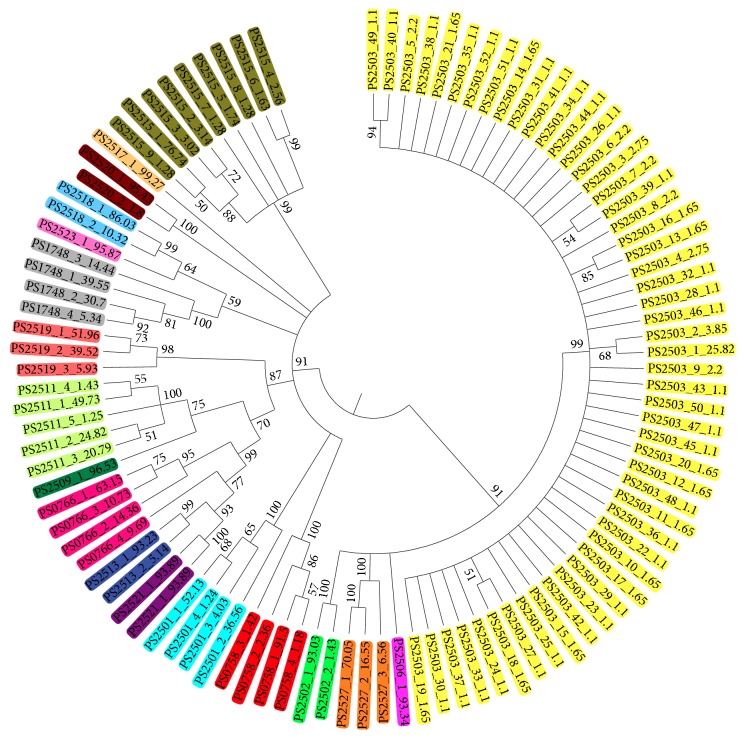
The neighbor-joining tree built using the major variants of ITS2 from 18* Dendrobium* species.* Note*. Each species was marked with a unique color. The three parts of the name of each branch represent the voucher number, the variant name of the multiple copy in the genome, and the percentage of its presence in the genome. The taxa with their voucher numbers were listed for the following:* D. williamsonii *(PS2503),* D. trigonopus *(PS2506),* D. acinaciforme *(PS2527),* D. capillipes *(PS2502),* D. loddigesii *(PS1748),* D. crystallinum *(PS2519),* D. denneanum *(PS0758),* D. chrysotoxum *(PS2501),* D. officinale *(PS2521),* D. gratiosissimum *(PS2513),* D. nobile *(PS0766),* D. wardianum *(PS2509),* D. pendulum *(PS2511),* D. aphyllum *(PS2523),* D. primulinum *(PS2518),* D. devonianum *(PS2520),* D. chrysanthum *(PS2515),* and D. crepidatum *(PS2517).

A neighbor-joining tree (NJ tree) was built based on the intragenomic data to determine the phylogenetic relationships between the* Dendrobium* species. Previous studies have shown that minor variants present below 1% are difficult to detect directly with PCR or clone sequencing. Thus, we first selected the major variants for analysis (Figure 2). The results showed that* D. williamsonii* (PS2503),* D. trigonopus* (PS2506),* D. acinaciforme* (PS2527), and* D. capillipes* (PS2502) clustered into one clade, with all other species forming a separate clade. Almost all the major variants clustered together, with the exception of* D. nobile*. One of the major variants of* D. nobile* (PS0766_4) introgressed into* D. officinale* and* D. gratiosissimum,* showing a very close relationship. Another NJ tree using total intragenomic data was also constructed and is shown in Appendix S3.

**Figure 3:**
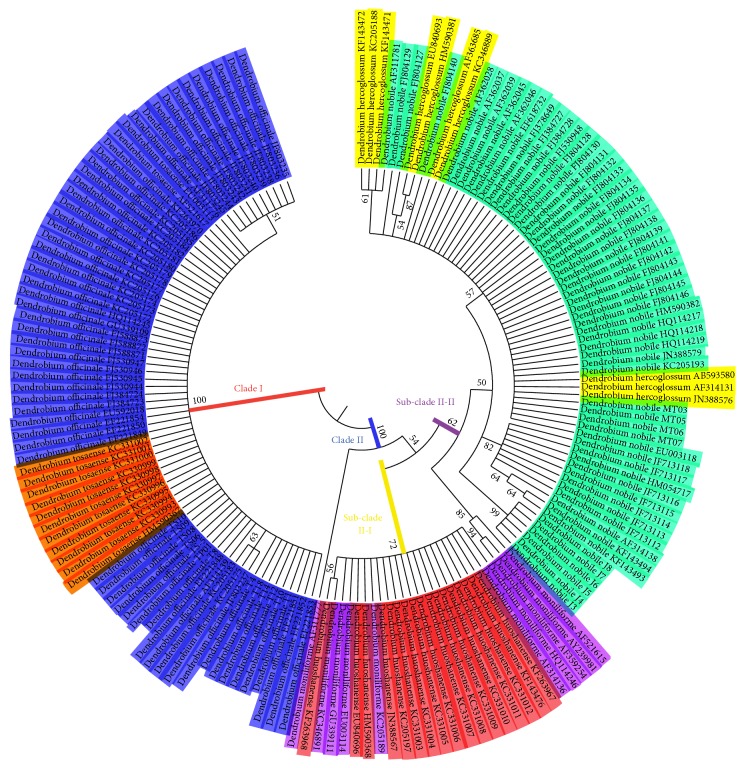
The neighbor-joining tree built using ITS2 sequences of the six species.* Note*. Different colors in Figure 3 represent different species. Blue,* D. officinale*; red,* D. huoshanense*; green,* D. nobile*; purple*, D. moniliforme*; orange,* D. tosaense*; yellow,* D. hercoglossum*.

Total intraspecific data were also used to construct an NJ tree for phylogenetic analysis (see Appendix S4). The results showed that most species were monophyletic except for one clade including six species (*D. officinale*,* D. tosaense*,* D. huoshanense*,* D. moniliforme*,* D. nobile,* and* D. hercoglossum*). To better clarify the relationship among these species in the main clade, these six species were used to build a separate NJ tree based on their ITS2 sequences (Figure 3). We divided this NJ tree into two major clades (Clades I and II). Clade I consists of two species,* D. officinale* and* D. tosaense*, with a bootstrap support value of 100%. Clade II consists of two subclades, with a bootstrap support value of 54%. Subclades II-I contain two species,* D. moniliforme* and* D. huoshanense*, with a bootstrap support value of 72%, and subclades II-II contain three species,* D. moniliforme*,* D. hercoglossum,* and* D. nobile*, with a bootstrap support value of 62%.

## 4. Discussion

Ribosomal DNA (rDNA) is present in multiple copies of tandem repeats per genome [31], and two noncoding spacers (internal transcribed spacer 1 and 2) divide each transcriptional unit into three subunits: 18S, 5.8S and 28S. Each tandem can contain variations, thus leading to intragenomic variation. Many studies have addressed genomic divergence in* Dendrobium*, but most of these have been focused on intra- and interspecific levels of variation [32–36]. It is thought that biodiversity at the species level is generally overestimated due to intragenomic variation [37]. In this study we therefore focused on the intragenomic level, aiming to identify relationships between intragenomic diversity and intraspecific diversity. Sequence-based methods have replaced many traditional approaches such as allozyme or restriction enzyme polymorphisms, which is valid as long as appropriate marker(s) is selected [38]. Traditional approaches (e.g., RAPD, AP-PCR, and AFLP) generally require high-quality DNA for amplification, which can lead to problems with reproducibility and accuracy. Sequence-based methods should be more objective and stable, enhancing our ability to assess biodiversity and identify species [39]. In addition, experimental error and subjective factors such as scoring PCR bands on a gel are eliminated or minimized in sequence-based protocols.

The ITS2 locus has already been proposed as a universal DNA barcode, particularly in plants, and it has been shown that plants can be identified at the species and genus level with more than 97% accuracy [27, 40]. Although the China plant BOL group suggested ITS as the core barcode for seed plants, ITS2 has several advantages compared with the full-length ITS region [41]. First, ITS2 is shorter than ITS, which simplifies PCR amplification. Moreover, ITS2 has secondary structure in all eukaryotes [42, 43]. This molecular morphological characteristic strengthens the power of its discriminatory ability. In addition to species identification applications, ITS2 and its secondary structure have been used as effective tools for phylogenetic analyses in insects, corals, and yeast [44–47]. As these transcribed spacers are highly divergent, they can also be used to estimate low levels of genetic diversity among related species [48]. Liu et al. evaluated the resolution of five regions (*rbcL*,* matK*, ITS, ITS2, and* trnH-psbA*), ultimately suggesting an* rbcL* + ITS2 barcode combination as the most suitable marker for analyzing biodiversity in the Dinghushan National Nature Reserve (DNNR) in China [49].

Among all the* Dendrobium* species,* D. officinale* is undoubtedly the most valuable, owing to its low production but high price and clinical efficacy in the clinic. Previous studies using ISSR, RAPD, and SRAP revealed distinct genetic differences and extensive genetic diversity among different populations of* D. officinale *[34, 50, 51]. However, the intraspecific genetic diversity of* D. officinale* (intraspecific genetic distance, average: 0.001; Max: 0.013) as revealed by ITS2 sequences turned out to be relatively low compared with results from other approaches. From the 61 ITS2 sequences obtained from* D. officinale*, only five variable sites were detected after alignment. Across the whole genome,* D. officinale* has a single dominant variant that represents 93.89% of ITS2 sequences. These results indicate that the ITS2 regions are relatively conserved among different populations of* D. officinale*. Due to the low production and high price of* D. officinale*, there are so many closely related species appearing as adulterants in the herbal market. These adulterants are species that have morphological characteristics similar to each other, making traditional taxonomic identification difficult, particularly after processing into medicinal slices. According to this result above, ITS2 can be an effective molecules tool for identifying commercial* D. officinale* and other* Dendrobium *species.

In the Chinese Pharmacopoeia (2015 edition),* D. officinale* is described as an independent species that is the source of the herbal medicine “Tie Pi Shi Hu.” However, this species has already been accepted as a synonym of* D. catenatum*,* D. tosaense*, and several others in flora of China and the other research [52]. In our study, ITS2 sequences from these two species were grouped into a single clade with 100% bootstrap support. The NJ tree described here demonstrates that, at the very least,* D. officinale* and* D. tosaense* are extremely closely related at the genetic level, consistent with other results from China. Therefore, we agree that* D. officinale* and* D. tosaense* should be accepted as synonyms of* D. catenatum*. In a previous study, a phylogenetic tree including twelve samples of* Dendrobium* species was constructed [50]. The three species* D. moniliforme*,* D. hercoglossum,* and* D. nobile* were grouped in the same clade, similar to classifications based on inflorescence color and the results from this study.

## 5. Conclusion

In this study, we analyzed intragenome and intraspecies divergence to find that, in most cases, greater intragenomic divergence is correlated with larger intraspecific variation. The results of this study strongly confirm that the direct PCR sequencing data were credible because all the dominant sequences in high-throughput sequencing in each species were detected by direct PCR. Thus, the multiple copies in ITS2 did not affect the species identification in* Dendrobium.* Therefore, we demonstrate that ITS2 is an effective tool for* Dendrobium* species identification.

## Supplementary Material

Appendix S1: Sampling information of this study. Appendix S2: GenBank sequences used in this study. Appendix S3: Neighbor-Joining tree using total intra-genomic data. Appendix S4: Neighbor-Joining tree using total intra-specific data for phylogenetic analysis.

## Figures and Tables

**Table 1:** Intragenomic diversity analysis of ITS2 major variants from the 18 *Dendrobium *species.

Taxon	Number of total 454 sequences	Number of total variants	Variant name (>1% emergence frequency)	Percentage of each variant (>1%)	Length (bp)	G + C content (%)	Intragenomic sequence distances (>1% emergence frequency)
*D. crepidatum*	1919	4	p1-1	99.27	248	50.81	0

*D. aphyllum*	1888	19	p2-1	95.87	248	52.42	0

*D. devonianum*	722	8	p3-1	95.43	248	49.20	0.004
			p3-2	1.80	247	48.58	

*D. officinale*	2554	10	p4-1	93.89	246	50.81	0^*∗*^
			p4-2	4.78	245	51.02	

*D. denneanum*	1270	15	p5-1	91.50	248	51.21	0.016
			p5-2	2.36	248	50.81	
			p5-3	1.42	248	50.00	
			p5-4	1.18	248	50.81	

*D. nobile*	578	7	p6-1	63.15	246	53.66	0.017
			p6-2	14.36	246	54.07	
			p6-3	10.73	246	54.07	
			p6-4	9.69	246	52.85	

*D. chrysanthum*	860	30	p7-1	76.74	245	52.24	0.051
			p7-2	3.14	245	51.84	
			p7-3	3.02	244	52.05	
			p7-4	2.56	245	47.35	
			p7-5	1.74	245	48.16	
			p7-6	1.63	245	47.76	
			p7-7	1.28	245	48.57	
			p7-8	1.28	245	50.20	
			p7-9	1.28	244	52.46	

*D. primulinum*	3690	36	p8-1	75.93	248	53.63	0.005
			p8-2	9.51	248	53.23	
			p8-3	9.11	248	53.23	

*D. chrysotoxum*	2366	55	p9-1	63.82	249	55.42	0.081
			p9-2	19.95	249	55.82	
			p9-3	2.79	249	55.02	
			p9-4	2.20	247	52.63	
			p9-5	2.20	249	55.42	
			p9-6	1.65	247	53.04	

*D. loddigesii*	1593	47	p10-1	39.55	248	52.02	0.009
			p10-2	30.70	248	51.21	
			p10-3	14.44	248	52.42	
			p10-4	5.34	248	51.21	

*D. acinaciforme*	1112	30	p11-1	70.05	248	52.42	0.045
			p11-2	16.55	248	52.42	
			p11-3	6.56	248	53.23	

*D. williamsonii*	182	52	p12-1	25.82	247	52.63	0.05
			p12-2	3.85	247	52.23	
			p12-3	2.75	247	50.20	
			p12-4	3.75	247	51.01	
			p12-5	2.20	247	51.01	
			p12-6	2.20	247	51.82	
			p12-7	2.20	247	50.20	
			p12-8	2.20	247	48.99	
			p12-9	2.20	247	50.61	
			p12-10~p12-21	19.80	247–250	48.99–51.42	
			p12-22~p12-52	34.10	244–247	48.37–52.63	

*D. capillipes*	1610	33	p13-1	93.03	251	50.20	0.004
			p13-2	1.43	251	49.80	

*D. crystallinum*	1197	14	p14-1	51.96	250	55.60	0.005
			p14-2	39.52	250	55.60	
			p14-3	5.93	250	55.20	

*D. gratiosissimum*	1167	8	p15-1	93.23	246	52.85	0.004
			p15-2	5.14	246	52.44	

*D. trigonopus*	1906	27	p16-1	93.34	251	51.00	0

*D. wardianum*	2778	21	p17-1	84.38	250	56.00	0^*∗*^
			p17-2	12.35	251	55.78	

*D. pendulum*	2232	19	p18-1	49.73	250	54.00	0.005
			p18-2	24.82	250	53.20	
			p18-3	20.79	249	53.41	
			p18-4	1.43	250	53.60	
			p18-5	1.25	250	53.60	

^*∗*^The variations were caused by insert/deletion, which was treated as complete deletion when calculating the K2P distances. Thus, the K2P distances turned out to be zero.

**Table 2:** Comparison between the numbers of intraspecific variable sites with the emergency percentage of the dominant variants in genome of ITS2 sequences of each species.

Taxon	Number of total ITS2 sequences	Number of total intraspecific variable sites	The emergency percentage of the dominant variants in genome (%)
*D. crepidatum*	35	36	99.27
*D. aphyllum*	22	4	95.87
*D. devonianum*	11	9	95.43
*D. officinale*	61	5	93.89
*D. denneanum*	10	4	91.50
*D. chrysanthum*	25	22	76.74
*D. primulinum*	21	25	75.93
*D. chrysotoxum*	27	18	63.82
*D. nobile*	59	12	63.15
*D. loddigesii*	13	28	39.55

## Abbreviations

ITS2: Internal transcribed spacer 2
rDNA: Ribosomal DNA
IS-GD: Intraspecific genetic distances
ITS: Internal transcribed spacer
IG-GD: Intragenomic genetic distance
SNP: Single nucleotide polymorphisms
HMM: Hidden Markov model
NJ tree: Neighbor-joining tree
PCR: Polymerase chain reaction
bp: Base pair
INDELS: Insertions and deletions
RAPD: Random amplified polymorphic DNA
AP-PCR: Arbitrarily primed PCR
AFLP: Amplified fragment length polymorphism
matK: Maturase K
rbcL: Ribulose-bisphosphate carboxylase gene
trnH-psbA: Intergenic spacer
ISSR: Inter-simple sequence repeat
SRAP: Sequence-related amplified polymorphism.

## Additional Points

*Supporting Information.* DNA sequences can be obtained through GenBank accessions; final DNA sequence assembly can be found in online supporting information.
