# Supplementary material for: Barcoding the Dendrobium (Orchidaceae) Species and Analysis of the Intragenomic Variation Based on the Internal Transcribed Spacer 2
Source: Biomed Res Int. 2017 Oct 17;2017:2734960. doi: 10.1155/2017/2734960 (PMC5664236; doi:10.1155/2017/2734960)
Supplement: Supplementary file 1 — Appendix S1: Sampling information of this study. Appendix S2: GenBank sequences used in this study. Appendix S3: Neighbor-Joining tree using total intra-genomic data. Appendix S4: Neighbor-Joining tree using total intra-specific data for phylogenetic analysis. [file 2734960.f1.zip › 2734960.f1/Appendix S1 Sampling information of this study.docx]

Appendix S1 Sampling information of this study

| Taxon | Sample Name | Sampling Sources |
| --- | --- | --- |
| *Dendrobium_chrysotoxum* | A-3 | Purchased online |
| *Dendrobium_chrysotoxum* | B-51 | Purchased online |
| *Dendrobium_chrysotoxum* | G1 | Purchased online |
| *Dendrobium_chrysotoxum* | G2 | Purchased online |
| *Dendrobium_chrysotoxum* | G3 | Purchased online |
| *Dendrobium_chrysotoxum* | G4 | Purchased online |
| *Dendrobium_chrysotoxum* | G6 | Purchased online |
| *Dendrobium_chrysotoxum* | MT01 | Botanical Garden of Xishuangbanna South Medicine |
| *Dendrobium_chrysotoxum* | MT02 | Botanical Garden of Xishuangbanna South Medicine |
| *Dendrobium_chrysotoxum* | MT03 | Botanical Garden of Xishuangbanna South Medicine |
| *Dendrobium_chrysotoxum* | MT05 | Botanical Garden of Xishuangbanna South Medicine |
| *Dendrobium_fimbriatum* | L1 | Purchased online |
| *Dendrobium_fimbriatum* | L2 | Purchased online |
| *Dendrobium_fimbriatum* | L3 | Purchased online |
| *Dendrobium_fimbriatum* | L6 | Purchased online |
| *Dendrobium_fimbriatum* | L7 | Purchased online |
| *Dendrobium_fimbriatum* | L8 | Purchased online |
| *Dendrobium_fimbriatum* | MT01 | Botanical Garden of Xishuangbanna South Medicine |
| *Dendrobium_fimbriatum* | MT04 | Ya'an, Sichuan |
| *Dendrobium_nobile* | J3 | Purchased online |
| *Dendrobium_nobile* | J4 | Purchased online |
| *Dendrobium_nobile* | J5 | Purchased online |
| *Dendrobium_nobile* | J6 | Purchased online |
| *Dendrobium_nobile* | J7 | Purchased online |
| *Dendrobium_nobile* | J8 | Purchased online |
| *Dendrobium_nobile* | MT03 | Botanical Garden of Xishuangbanna South Medicine |
| *Dendrobium_nobile* | MT05 | Botanical Garden of Xishuangbanna South Medicine |
| *Dendrobium_nobile* | MT06 | Botanical Garden of Xishuangbanna South Medicine |
| *Dendrobium_nobile* | MT07 | Yulin, Guangxi |
| *Dendrobium_officinale* | T1 | Purchased online |
| *Dendrobium_officinale* | T10 | Purchased online |
| *Dendrobium_officinale* | T12 | Purchased online |
| *Dendrobium_officinale* | T14 | Purchased online |
| *Dendrobium_officinale* | T18 | Purchased online |
| *Dendrobium_officinale* | T4 | Purchased online |
| *Dendrobium_officinale* | T8 | Purchased online |
| *Dendrobium_officinale* | TP01 | Purchased online |
| *Dendrobium_officinale* | TS06 | Purchased online |
| *Dendrobium_officinale* | TS08 | Purchased online |
| *Dendrobium_officinale* | TS09 | Purchased online |
| *Dendrobium_officinale* | TS10 | Purchased online |
| *Dendrobium_officinale* | TS-5 | Purchased online |
