# Supplementary material for: Barcoding the Dendrobium (Orchidaceae) Species and Analysis of the Intragenomic Variation Based on the Internal Transcribed Spacer 2
Source: Biomed Res Int. 2017 Oct 17;2017:2734960. doi: 10.1155/2017/2734960 (PMC5664236; doi:10.1155/2017/2734960)
Supplement: Supplementary file 1 — Appendix S1: Sampling information of this study. Appendix S2: GenBank sequences used in this study. Appendix S3: Neighbor-Joining tree using total intra-genomic data. Appendix S4: Neighbor-Joining tree using total intra-specific data for phylogenetic analysis. [file 2734960.f1.zip › 2734960.f1/Appendix S2 GenBank sequences used in this study.docx]

Table S2 GenBank sequences used in this study

| GenBank Accession No. | GenBank Accession No. |
| --- | --- |
| *Dendrobium_amoenum_HM054534* | *Dendrobium_huoshanense_KC331008* |
| *Dendrobium_amoenum_HM054535* | *Dendrobium_huoshanense_KC331009* |
| *Dendrobium_amoenum_HM054536* | *Dendrobium_huoshanense_KC331010* |
| *Dendrobium_amoenum_HM054537* | *Dendrobium_huoshanense_KC331011* |
| *Dendrobium_amoenum_HM054538* | *Dendrobium_huoshanense_KC331012* |
| *Dendrobium_amoenum_HM054539* | *Dendrobium_huoshanense_KF143476* |
| *Dendrobium_amoenum_HM054540* | *Dendrobium_huoshanense_KF263967* |
| *Dendrobium_amoenum_HM054541* | *Dendrobium_huoshanense_KF263968* |
| *Dendrobium_amoenum_HM054542* | *Dendrobium_jenkinsii_AF314132* |
| *Dendrobium_amoenum_HM054543* | *Dendrobium_jenkinsii_DQ058785* |
| *Dendrobium_amoenum_HM054544* | *Dendrobium_jenkinsii_HM054670* |
| *Dendrobium_amoenum_HM054545* | *Dendrobium_jenkinsii_HM054671* |
| *Dendrobium_amoenum_HM054546* | *Dendrobium_jenkinsii_HQ114251* |
| *Dendrobium_aphyllum_AB593539* | *Dendrobium_jenkinsii_JF713108* |
| *Dendrobium_aphyllum_AF355573* | *Dendrobium_jenkinsii_JF713109* |
| *Dendrobium_aphyllum_EU840691* | *Dendrobium_jenkinsii_JN388595* |
| *Dendrobium_aphyllum_FJ428219* | *Dendrobium_jenkinsii_KF143478* |
| *Dendrobium_aphyllum_HM054549* | *Dendrobium_jenkinsii_KF143479* |
| *Dendrobium_aphyllum_HM054550* | *Dendrobium_loddigesii_AB593604* |
| *Dendrobium_aphyllum_HM054551* | *Dendrobium_loddigesii_AF311778* |
| *Dendrobium_aphyllum_HM054552* | *Dendrobium_loddigesii_AF314134* |
| *Dendrobium_aphyllum_HM054553* | *Dendrobium_loddigesii_AY485703* |
| *Dendrobium_aphyllum_HM054554* | *Dendrobium_loddigesii_EU121418* |
| *Dendrobium_aphyllum_HM054555* | *Dendrobium_loddigesii_EU592016* |
| *Dendrobium_aphyllum_HM054556* | *Dendrobium_loddigesii_HM590374* |
| *Dendrobium_aphyllum_HM054557* | *Dendrobium_loddigesii_HQ114220* |
| *Dendrobium_aphyllum_HM054558* | *Dendrobium_loddigesii_JN388569* |
| *Dendrobium_aphyllum_HM054559* | *Dendrobium_loddigesii_KC205187* |
| *Dendrobium_aphyllum_HM054560* | *Dendrobium_loddigesii_KF143481* |
| *Dendrobium_aphyllum_HM054561* | *Dendrobium_loddigesii_KJ658320* |
| *Dendrobium_aphyllum_HM590384* | *Dendrobium_loddigesii_KP159301* |
| *Dendrobium_aphyllum_HQ114247* | *Dendrobium_macrostachyum_HM054674* |
| *Dendrobium_aphyllum_HQ114248* | *Dendrobium_macrostachyum_HM054675* |
| *Dendrobium_aphyllum_JN388571* | *Dendrobium_macrostachyum_HM054676* |
| *Dendrobium_aphyllum_KF143430* | *Dendrobium_macrostachyum_HM054677* |
| *Dendrobium_aqueum_HM054562* | *Dendrobium_macrostachyum_HM054678* |
| *Dendrobium_aqueum_HM054563* | *Dendrobium_macrostachyum_HM054679* |
| *Dendrobium_aqueum_HM054564* | *Dendrobium_macrostachyum_HM054680* |
| *Dendrobium_aqueum_HM054565* | *Dendrobium_macrostachyum_HM054681* |
| *Dendrobium_aqueum_HM054566* | *Dendrobium_macrostachyum_HM054682* |
| *Dendrobium_aqueum_HM054567* | *Dendrobium_macrostachyum_HM054683* |
| *Dendrobium_aqueum_HM054568* | *Dendrobium_macrostachyum_HM054684* |
| *Dendrobium_aqueum_HM054569* | *Dendrobium_macrostachyum_HM054685* |
| *Dendrobium_aqueum_HM054570* | *Dendrobium_macrostachyum_HM054686* |
| *Dendrobium_aqueum_HM054571* | *Dendrobium_macrostachyum_HM054687* |
| *Dendrobium_aqueum_JF713085* | *Dendrobium_macrostachyum_HM054688* |
| *Dendrobium_aurantiacum_var._denneanum_AF362043* | *Dendrobium_macrostachyum_HM054689* |
| *Dendrobium_aurantiacum_var._denneanum_EU840702* | *Dendrobium_macrostachyum_HM054690* |
| *Dendrobium_aurantiacum_var._denneanum_FJ384729* | *Dendrobium_macrostachyum_HM054691* |
| *Dendrobium_aurantiacum_var._denneanum_FJ384730* | *Dendrobium_macrostachyum_HM054692* |
| *Dendrobium_aurantiacum_var._denneanum_FJ384732* | *Dendrobium_macrostachyum_HM054693* |
| *Dendrobium_aurantiacum_var._denneanum_FJ530949* | *Dendrobium_macrostachyum_HM054694* |
| *Dendrobium_aurantiacum_var._denneanum_GU339113* | *Dendrobium_macrostachyum_HM054695* |
| *Dendrobium_aurantiacum_var._denneanum_JN388572* | *Dendrobium_macrostachyum_HM054696* |
| *Dendrobium_aurantiacum_var._denneanum_KF143448* | *Dendrobium_macrostachyum_HM054697* |
| *Dendrobium_aurantiacum_var._denneanum_KF143450* | *Dendrobium_macrostachyum_HM054698* |
| *Dendrobium_barbatulum_HM054572* | *Dendrobium_macrostachyum_HM054699* |
| *Dendrobium_barbatulum_HM054573* | *Dendrobium_moniliforme_AF311777* |
| *Dendrobium_barbatulum_HM054574* | *Dendrobium_moniliforme_AF314136* |
| *Dendrobium_barbatulum_HM054575* | *Dendrobium_moniliforme_AF359254* |
| *Dendrobium_barbatulum_HM054576* | *Dendrobium_moniliforme_AF521615* |
| *Dendrobium_barbatulum_HM054577* | *Dendrobium_moniliforme_AY239981* |
| *Dendrobium_barbatulum_HM054578* | *Dendrobium_moniliforme_EU003114* |
| *Dendrobium_barbatulum_JF713086* | *Dendrobium_moniliforme_GU339111* |
| *Dendrobium_barbatulum_JF713087* | *Dendrobium_moniliforme_HQ114246* |
| *Dendrobium_barbatulum_JF713088* | *Dendrobium_moniliforme_KC205189* |
| *Dendrobium_bicameratum_HM054581* | *Dendrobium_moniliforme_KC346891* |
| *Dendrobium_bicameratum_HM054582* | *Dendrobium_moschatum_AF314137* |
| *Dendrobium_bicameratum_HM054583* | *Dendrobium_moschatum_AY239983* |
| *Dendrobium_bicameratum_HM054584* | *Dendrobium_moschatum_EF629326* |
| *Dendrobium_bicameratum_HM054585* | *Dendrobium_moschatum_HM054709* |
| *Dendrobium_bicameratum_HM054586* | *Dendrobium_moschatum_HM054710* |
| *Dendrobium_bicameratum_HM054587* | *Dendrobium_moschatum_HM054711* |
| *Dendrobium_bicameratum_HM054588* | *Dendrobium_moschatum_HM054712* |
| *Dendrobium_bicameratum_HM054589* | *Dendrobium_moschatum_HM054713* |
| *Dendrobium_bicameratum_HM054590* | *Dendrobium_moschatum_HM054714* |
| *Dendrobium_bicameratum_HM054591* | *Dendrobium_moschatum_HM054715* |
| *Dendrobium_chrysanthum_AF314126* | *Dendrobium_moschatum_HM054716* |
| *Dendrobium_chrysanthum_AF355572* | *Dendrobium_moschatum_JF713111* |
| *Dendrobium_chrysanthum_AF362047* | *Dendrobium_nobile_AF311781* |
| *Dendrobium_chrysanthum_EU003119* | *Dendrobium_nobile_AF314138* |
| *Dendrobium_chrysanthum_FJ384738* | *Dendrobium_nobile_AF362028* |
| *Dendrobium_chrysanthum_HM054592* | *Dendrobium_nobile_AF362037* |
| *Dendrobium_chrysanthum_HM054593* | *Dendrobium_nobile_AF362039* |
| *Dendrobium_chrysanthum_HM054594* | *Dendrobium_nobile_AF362045* |
| *Dendrobium_chrysanthum_HM054595* | *Dendrobium_nobile_AF362046* |
| *Dendrobium_chrysanthum_HM054596* | *Dendrobium_nobile_EF618732* |
| *Dendrobium_chrysanthum_HM054597* | *Dendrobium_nobile_EU003118* |
| *Dendrobium_chrysanthum_HM054598* | *Dendrobium_nobile_FJ378649* |
| *Dendrobium_chrysanthum_HM054599* | *Dendrobium_nobile_FJ384727* |
| *Dendrobium_chrysanthum_HM590376* | *Dendrobium_nobile_FJ384728* |
| *Dendrobium_chrysanthum_HQ114238* | *Dendrobium_nobile_FJ530948* |
| *Dendrobium_chrysanthum_JF713089* | *Dendrobium_nobile_FJ804127* |
| *Dendrobium_chrysanthum_JF713090* | *Dendrobium_nobile_FJ804128* |
| *Dendrobium_chrysanthum_JF713091* | *Dendrobium_nobile_FJ804129* |
| *Dendrobium_chrysanthum_JF713092* | *Dendrobium_nobile_FJ804130* |
| *Dendrobium_chrysanthum_JF713093* | *Dendrobium_nobile_FJ804131* |
| *Dendrobium_chrysanthum_JF713094* | *Dendrobium_nobile_FJ804132* |
| *Dendrobium_chrysanthum_JN388584* | *Dendrobium_nobile_FJ804133* |
| *Dendrobium_chrysanthum_KF143443* | *Dendrobium_nobile_FJ804134* |
| *Dendrobium_chrysanthum_KF263964* | *Dendrobium_nobile_FJ804135* |
| *Dendrobium_chrysanthum_KF263965* | *Dendrobium_nobile_FJ804136* |
| *Dendrobium_chrysotoxum_AB593533* | *Dendrobium_nobile_FJ804137* |
| *Dendrobium_chrysotoxum_AF314127* | *Dendrobium_nobile_FJ804138* |
| *Dendrobium_chrysotoxum_AF362023* | *Dendrobium_nobile_FJ804139* |
| *Dendrobium_chrysotoxum_EU477501* | *Dendrobium_nobile_FJ804140* |
| *Dendrobium_chrysotoxum_FJ384736* | *Dendrobium_nobile_FJ804141* |
| *Dendrobium_chrysotoxum_HM054600* | *Dendrobium_nobile_FJ804142* |
| *Dendrobium_chrysotoxum_HM054601* | *Dendrobium_nobile_FJ804143* |
| *Dendrobium_chrysotoxum_HM054602* | *Dendrobium_nobile_FJ804144* |
| *Dendrobium_chrysotoxum_HM590383* | *Dendrobium_nobile_FJ804145* |
| *Dendrobium_chrysotoxum_HQ114221* | *Dendrobium_nobile_FJ804146* |
| *Dendrobium_chrysotoxum_HQ114222* | *Dendrobium_nobile_HM054717* |
| *Dendrobium_chrysotoxum_HQ114223* | *Dendrobium_nobile_HM590382* |
| *Dendrobium_chrysotoxum_JN388585* | *Dendrobium_nobile_HQ114217* |
| *Dendrobium_chrysotoxum_KC413407* | *Dendrobium_nobile_HQ114218* |
| *Dendrobium_chrysotoxum_KF143444* | *Dendrobium_nobile_HQ114219* |
| *Dendrobium_chrysotoxum_KJ658324* | *Dendrobium_nobile_JF713112* |
| *Dendrobium_crepidatum_AB593534* | *Dendrobium_nobile_JF713113* |
| *Dendrobium_crepidatum_AF314128* | *Dendrobium_nobile_JF713114* |
| *Dendrobium_crepidatum_AF355574* | *Dendrobium_nobile_JF713115* |
| *Dendrobium_crepidatum_AY842035* | *Dendrobium_nobile_JF713116* |
| *Dendrobium_crepidatum_HM054603* | *Dendrobium_nobile_JF713117* |
| *Dendrobium_crepidatum_HM054604* | *Dendrobium_nobile_JF713118* |
| *Dendrobium_crepidatum_HM054605* | *Dendrobium_nobile_JN388579* |
| *Dendrobium_crepidatum_HM054606* | *Dendrobium_nobile_KC205193* |
| *Dendrobium_crepidatum_HM054607* | *Dendrobium_nobile_KF143493* |
| *Dendrobium_crepidatum_HM054608* | *Dendrobium_nobile_KF143494* |
| *Dendrobium_crepidatum_HM054609* | *Dendrobium_officinale_AF311776* |
| *Dendrobium_crepidatum_HM054610* | *Dendrobium_officinale_AF314139* |
| *Dendrobium_crepidatum_HM054611* | *Dendrobium_officinale_EF221848* |
| *Dendrobium_crepidatum_HM054612* | *Dendrobium_officinale_EF221849* |
| *Dendrobium_crepidatum_HM054613* | *Dendrobium_officinale_EF221850* |
| *Dendrobium_crepidatum_HM054614* | *Dendrobium_officinale_EF221851* |
| *Dendrobium_crepidatum_HM054615* | *Dendrobium_officinale_EF221852* |
| *Dendrobium_crepidatum_HM054616* | *Dendrobium_officinale_EF221853* |
| *Dendrobium_crepidatum_HM054617* | *Dendrobium_officinale_EF221854* |
| *Dendrobium_crepidatum_HM054618* | *Dendrobium_officinale_EU592018* |
| *Dendrobium_crepidatum_HM054619* | *Dendrobium_officinale_FJ384723* |
| *Dendrobium_crepidatum_HM054620* | *Dendrobium_officinale_FJ384724* |
| *Dendrobium_crepidatum_HM054621* | *Dendrobium_officinale_FJ530944* |
| *Dendrobium_crepidatum_HM054622* | *Dendrobium_officinale_FJ530945* |
| *Dendrobium_crepidatum_HM054623* | *Dendrobium_officinale_FJ530946* |
| *Dendrobium_crepidatum_HM054624* | *Dendrobium_officinale_FJ530947* |
| *Dendrobium_crepidatum_HQ114240* | *Dendrobium_officinale_FJ588871* |
| *Dendrobium_crepidatum_JN388586* | *Dendrobium_officinale_FJ588872* |
| *Dendrobium_crepidatum_KF143446* | *Dendrobium_officinale_FJ588873* |
| *Dendrobium_crepidatum_KJ210430* | *Dendrobium_officinale_GU339109* |
| *Dendrobium_crepidatum_KJ210431* | *Dendrobium_officinale_HQ114245* |
| *Dendrobium_crepidatum_KJ210432* | *Dendrobium_officinale_JF803235* |
| *Dendrobium_crepidatum_KJ210433* | *Dendrobium_officinale_JF803236* |
| *Dendrobium_crepidatum_KJ658319* | *Dendrobium_officinale_JF803237* |
| *Dendrobium_crepidatum_KJ944628* | *Dendrobium_officinale_JF803238* |
| *Dendrobium_devonianum_AB593548* | *Dendrobium_officinale_JF803239* |
| *Dendrobium_devonianum_AF311779* | *Dendrobium_officinale_JF803240* |
| *Dendrobium_devonianum_EU477502* | *Dendrobium_officinale_JF803241* |
| *Dendrobium_devonianum_FJ384735* | *Dendrobium_officinale_JF803242* |
| *Dendrobium_devonianum_HQ114244* | *Dendrobium_officinale_JF803243* |
| *Dendrobium_devonianum_JF713098* | *Dendrobium_officinale_JF803244* |
| *Dendrobium_devonianum_JF713099* | *Dendrobium_officinale_JF803245* |
| *Dendrobium_devonianum_JF713100* | *Dendrobium_officinale_KC205172* |
| *Dendrobium_devonianum_KC205194* | *Dendrobium_officinale_KC205173* |
| *Dendrobium_devonianum_KC346888* | *Dendrobium_officinale_KC205174* |
| *Dendrobium_devonianum_KF143453* | *Dendrobium_officinale_KC205175* |
| *Dendrobium_fimbriatum_AB593562* | *Dendrobium_officinale_KC205176* |
| *Dendrobium_fimbriatum_AF314130* | *Dendrobium_officinale_KC205177* |
| *Dendrobium_fimbriatum_AY842036* | *Dendrobium_officinale_KC205178* |
| *Dendrobium_fimbriatum_EU003116* | *Dendrobium_officinale_KC205179* |
| *Dendrobium_fimbriatum_HM054632* | *Dendrobium_officinale_KC205180* |
| *Dendrobium_fimbriatum_HM054634* | *Dendrobium_officinale_KC205181* |
| *Dendrobium_fimbriatum_HM054635* | *Dendrobium_officinale_KC205182* |
| *Dendrobium_fimbriatum_HM054636* | *Dendrobium_officinale_KC205183* |
| *Dendrobium_fimbriatum_HM054637* | *Dendrobium_officinale_KC205184* |
| *Dendrobium_fimbriatum_HM590392* | *Dendrobium_officinale_KC205185* |
| *Dendrobium_fimbriatum_HQ114229* | *Dendrobium_officinale_KC205186* |
| *Dendrobium_fimbriatum_JN388588* | *Dendrobium_officinale_KF263970* |
| *Dendrobium_fimbriatum_KF143461* | *Dendrobium_ovatum_HM054721* |
| *Dendrobium_fimbriatum_KJ658308* | *Dendrobium_ovatum_HM054722* |
| *Dendrobium_hancockii_AB593575* | *Dendrobium_ovatum_HM054723* |
| *Dendrobium_hancockii_AF362025* | *Dendrobium_ovatum_HM054724* |
| *Dendrobium_hancockii_AF362038* | *Dendrobium_ovatum_HM054725* |
| *Dendrobium_hancockii_DQ058787* | *Dendrobium_ovatum_HM054726* |
| *Dendrobium_hancockii_EU003120* | *Dendrobium_ovatum_HM054727* |
| *Dendrobium_hancockii_FJ384725* | *Dendrobium_ovatum_HM054728* |
| *Dendrobium_hancockii_FJ384726* | *Dendrobium_ovatum_HM054729* |
| *Dendrobium_hancockii_HM590377* | *Dendrobium_ovatum_HM054730* |
| *Dendrobium_hancockii_HQ114259* | *Dendrobium_ovatum_HM054731* |
| *Dendrobium_hancockii_JN388591* | *Dendrobium_ovatum_HM054732* |
| *Dendrobium_hancockii_KF143467* | *Dendrobium_ovatum_HM054733* |
| *Dendrobium_hancockii_KF263966* | *Dendrobium_ovatum_HM054734* |
| *Dendrobium_herbaceum_HM054640* | *Dendrobium_peguanum_HM054737* |
| *Dendrobium_herbaceum_HM054641* | *Dendrobium_peguanum_HM054738* |
| *Dendrobium_herbaceum_HM054642* | *Dendrobium_peguanum_HM054739* |
| *Dendrobium_herbaceum_HM054643* | *Dendrobium_peguanum_HM054740* |
| *Dendrobium_herbaceum_HM054644* | *Dendrobium_peguanum_HM054741* |
| *Dendrobium_herbaceum_HM054645* | *Dendrobium_peguanum_HM054742* |
| *Dendrobium_herbaceum_HM054646* | *Dendrobium_peguanum_HM054743* |
| *Dendrobium_herbaceum_HM054647* | *Dendrobium_peguanum_HM054744* |
| *Dendrobium_herbaceum_HM054648* | *Dendrobium_peguanum_HM054745* |
| *Dendrobium_herbaceum_HM054649* | *Dendrobium_peguanum_HM054746* |
| *Dendrobium_herbaceum_HM054650* | *Dendrobium_primulinum_AB593641* |
| *Dendrobium_herbaceum_HM054651* | *Dendrobium_primulinum_AF362913* |
| *Dendrobium_herbaceum_HM054652* | *Dendrobium_primulinum_AY485715* |
| *Dendrobium_herbaceum_HM054653* | *Dendrobium_primulinum_HM054747* |
| *Dendrobium_herbaceum_HM054654* | *Dendrobium_primulinum_HM054748* |
| *Dendrobium_herbaceum_HM054655* | *Dendrobium_primulinum_HM054749* |
| *Dendrobium_herbaceum_HM054656* | *Dendrobium_primulinum_HM054750* |
| *Dendrobium_herbaceum_HM054657* | *Dendrobium_primulinum_HM054751* |
| *Dendrobium_herbaceum_HM054658* | *Dendrobium_primulinum_HM054752* |
| *Dendrobium_herbaceum_HM054659* | *Dendrobium_primulinum_HM054753* |
| *Dendrobium_herbaceum_HM054660* | *Dendrobium_primulinum_HM054754* |
| *Dendrobium_herbaceum_HM054661* | *Dendrobium_primulinum_HM054755* |
| *Dendrobium_herbaceum_HM054662* | *Dendrobium_primulinum_HM054756* |
| *Dendrobium_herbaceum_HM054663* | *Dendrobium_primulinum_HM054757* |
| *Dendrobium_herbaceum_HM054664* | *Dendrobium_primulinum_HQ114242* |
| *Dendrobium_herbaceum_HM054665* | *Dendrobium_primulinum_JF713123* |
| *Dendrobium_herbaceum_HM054666* | *Dendrobium_primulinum_JN388597* |
| *Dendrobium_hercoglossum_AB593580* | *Dendrobium_primulinum_JN388598* |
| *Dendrobium_hercoglossum_AF314131* | *Dendrobium_primulinum_KF143499* |
| *Dendrobium_hercoglossum_AF363685* | *Dendrobium_primulinum_KJ658309* |
| *Dendrobium_hercoglossum_EU840693* | *Dendrobium_primulinum_KJ944625* |
| *Dendrobium_hercoglossum_HM590381* | *Dendrobium_tosaense_AF401488* |
| *Dendrobium_hercoglossum_JN388576* | *Dendrobium_tosaense_AF521617* |
| *Dendrobium_hercoglossum_KC205188* | *Dendrobium_tosaense_EU003113* |
| *Dendrobium_hercoglossum_KC346889* | *Dendrobium_tosaense_HM590367* |
| *Dendrobium_hercoglossum_KF143471* | *Dendrobium_tosaense_KC330993* |
| *Dendrobium_hercoglossum_KF143472* | *Dendrobium_tosaense_KC330994* |
| *Dendrobium_huoshanense_EU840696* | *Dendrobium_tosaense_KC330995* |
| *Dendrobium_huoshanense_HM590368* | *Dendrobium_tosaense_KC330996* |
| *Dendrobium_huoshanense_JN388567* | *Dendrobium_tosaense_KC330997* |
| *Dendrobium_huoshanense_KC205197* | *Dendrobium_tosaense_KC330998* |
| *Dendrobium_huoshanense_KC331003* | *Dendrobium_tosaense_KC330999* |
| *Dendrobium_huoshanense_KC331004* | *Dendrobium_tosaense_KC331000* |
| *Dendrobium_huoshanense_KC331005* | *Dendrobium_tosaense_KC331001* |
| *Dendrobium_huoshanense_KC331006* | *Dendrobium_tosaense_KC331002* |
| *Dendrobium_huoshanense_KC331007* |  |
